# Sensory analysis of the flavor profile of full immersion hot, room temperature, and cold brewed coffee over time

**DOI:** 10.1038/s41598-024-69867-6

**Published:** 2024-08-20

**Authors:** Jiexin Liang, Mackenzie E. Batali, Catherine Routt, William D. Ristenpart, Jean-Xavier Guinard

**Affiliations:** 1 Department of Food Science and Technology, University of California, Davis, One Shields Avenue, Davis, CA 95616 USA; 2 Department of Chemical Engineering, University of California, Davis, One Shields Avenue, Davis, CA 95616 USA

**Keywords:** Plant sciences, Engineering

## Abstract

With the growing popularity of cold brewed coffee comes a need for brewing efficiency while preserving the desirable flavor profile. Despite the wide use of full immersion brewing techniques, the effect of brew time on the dynamic sensory profiles of full immersion brewed coffee remains underexplored. Here, we investigated the relationship between coffee sensory quality and extraction dynamics, measured as Total Dissolved Solids (*TDS*) and Extraction (*E*) of full immersion brewed coffee at various roast levels, and brewing temperatures (4 °C, 22 °C and 92 °C), over brew time using a generic descriptive analysis method. Specifically, different brew time points were selected for different temperatures based on five targeted coffee extraction stages. Furthermore, the unique experimental design also explored a sensory-driven engineering research process. Roast level had the greatest impact on the sensory profile of the coffees, followed by brewing temperature, but brew time, especially the longer brew times as *TDS* plateaued, had subtler impacts than expected. Twenty-five of 28 sensory attributes were significantly different among the 30 coffee samples, indicating a single source green coffee blend can produce a wide range of complex sensory profiles using different combinations of roast level, temperature, and brew time. Specifically, the intensity of sweetness was negatively correlated with *TDS*, and 19 other attribute intensities were positively correlated with *TDS*. Interestingly, we found that certain long time cold brews had similar sensory profiles to those of some short time hot brews, suggesting the sensory profiles of certain hot brews and cold brews could possibly be matched through controlled preparation. Overall, our study demonstrated an approach of integrating food engineering and sensory analysis for product development, and our findings provide valuable insights into the extraction dynamics and sensory quality of full immersion brewed coffee and opens new brewing avenues for the coffee industry.

## Introduction

The importance of *TDS* and *E* on sensory quality and consumer liking of coffee has been well established^1–5^. While most coffee sensory studies of brewed coffee focused on pressurized espresso brew or flow-based drip brew^2,4,6–10^, there are comparatively less sensory studies on full immersion brewing methods other than a few recent ones focused on cold brew^10–14^. The connection between sensory quality and extraction dynamics of full immersion brewed coffee under various brewing conditions is also missing. Especially, for coffee cupping, the coffee industry relies on the Specialty Coffee Association (SCA) standards and protocols as guidelines to prepare coffee samples^15^. However, the effects of brewing parameters on extraction dynamics in the cupping brewing process remain underexplored. The process of cupping involves tasting the coffee at multiple time points as coffee cools down throughout the entire brewing process^15^. However, no scientific study to date has systematically assessed the dynamic sensory profile behind full immersion brewed coffee in response to various brewing parameters, such as grind size, brew ratio, brew temperature, and roast level. Moreover, the difference in extraction dynamics in response to roast level also has crucial implications for sensory quality of full immersion brewed coffee: the sensory properties of coffees with different roast levels evaluated at the same time point under the same temperature could be confounded by the difference in strength^16,17^. Therefore, the sensory quality of full immersion brewed coffee depends on multiple brewing parameters.

To understand the effects of roast level and key brewing parameters on the sensory quality of the brew, a combination of engineering and sensory evaluation approaches is needed to investigate how physical characteristics of the cup vary with controllable parameters, and how such measurable physical properties (*TDS* and E) correlate with sensory properties of full immersion brewed coffee. For the vast majority of coffee baristas and coffee consumers, the practical question of how to brew a cup of coffee with a desired sensory profile using full immersion brewing techniques has yet to be fully answered.

Our prior sensory analysis of the effects of origin, roast level, and brewing temperature on the flavor profile of full immersion brewed coffee at fixed equilibrium *TDS* and cold consumption temperature showed that brew temperature across the cold to hot range has a significant impact on the sensory properties of coffee, and that different roast levels and origins also yield differences in sensory attributes with temperature^18^. Yet, this study eliminated the effect of total brew time that yields differences in final *TDS*, which is also a key factor in coffee cup quality^17,19^.

On the other hand, the coffee industry heavily relies on the full immersion method of “cupping” to evaluate coffee quality, mainly following SCA standards and protocols^15^. Despite the widespread use of this procedure and the industry’s continuous dedication to enhancing it^20^, questions remain about the consistency and effectiveness of this sensory evaluation process used in the industry^21–23^, and there is still a need for published scientific justification of the details of the protocol. We therefore wanted to answer the following questions: how does the sensory profile of the coffee cup change over time after water addition, as it continues to extract, and cools down to room temperature? How does this dynamic sensory profile change in response to different roast levels and brewing parameters? What are the relationships between the physicochemical measurements (*TDS*, pH, and TA) and the sensory quality of coffee over time? How do we design the sensory profile of immersion coffee via actionable brewing parameters?

To answer these questions, this study related the extraction dynamics to the sensory quality of full immersion coffee following a sensory-driven engineering approach. We explored the flavor profiles and investigated the effects of roast levels, brewing temperatures, and brew time on the sensory quality of full immersion brewed coffee using a generic descriptive analysis method in conjunction with *TDS*, TA, and pH measurements.

## Methods

### Experimental design

We used a 2 × 3 × 5 factorial design, with a Central America coffee blend roasted to 2 different roast levels, and each brewed at three different temperatures (4 °C, 22 °C, and 92 °C), to five different time points that corresponded to five brewing stages from “rinse” to equilibrium of full immersion brews. Each of the 30 coffee samples were prepared in triplicate for both physical/chemical and sensory measurements. The physical/chemical properties of the coffees were measured as total dissolved solids (*TDS*), titratable acidity (TA), and pH, and the sensory properties of the coffees were evaluated using a generic descriptive analysis method.

### Coffees

A Central American blend coffee composed of an El Salvador Cerro Las Ranas coffee and a Nicaragua Parainema coffee was roasted to two different roast levels, representing a “light” roast, denoted by light roast (L), and a darker end of “medium-dark” roast, denoted by dark roast (D)^24^. Roasting was done in a single day using a Probat Probatone P-5 roaster (Probat-Werkevon Gimborn Maschinenfabrik GmbH, Emmerich am Rhein, Germany) at the UC Davis Coffee Center. The average roast colors of the roasted beans were 71.8 and 41.8 for the light and dark roasts, respectively, measured using an Agtron Gourmet Color Scale^25^ (Agtron E2OCP-II Coffee Analyzer, Agtron Inc., Reno, NV, USA). Roasted beans were degassed for a week at room temperature, then vacuum-sealed in bags and stored in a freezer at − 20 °C following the same protocol as in previously published coffee studies^16,18^. To ensure consistent sensory quality, individual bags of coffee were defrosted at room temperature overnight before use.

### Coffee brewing

**Figure 1:**
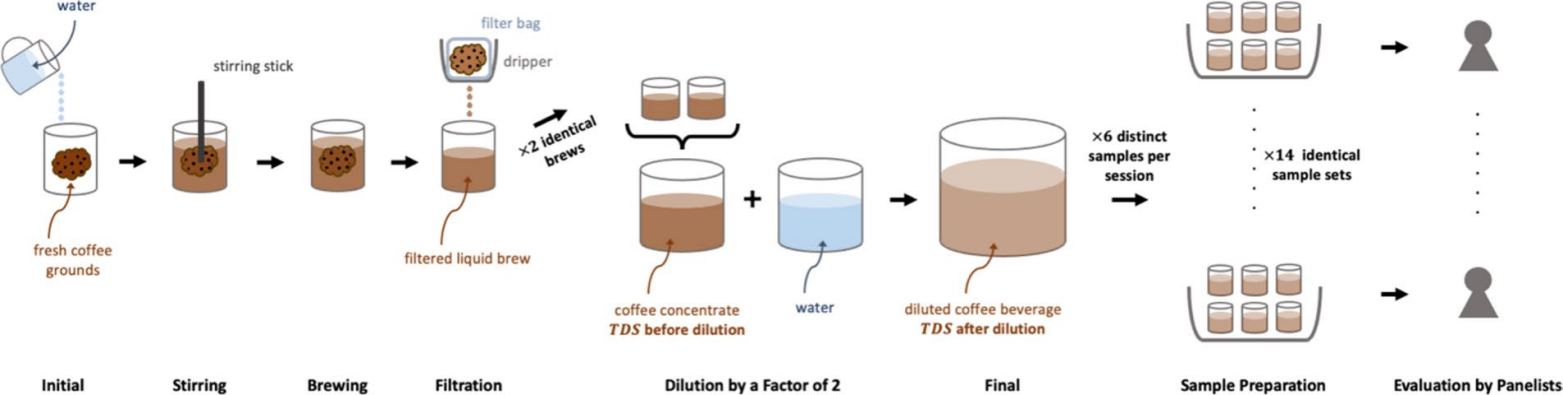
A schematic of coffee brewing and the sensory evaluation process.

A schematic visualization of the brewing and serving process is shown in Fig. 1. All roasted coffee beans were ground to a consistent grind size setting of 4, corresponding to a median particle size of $972\pm 19$ μm (cf. Supplemental Fig. S1 in^19^ reference) in a classic Mahlkönig Guatemala Lab Grinder (Mahlkönig USA, Durham, NC, USA). Coffees from each roast level were brewed using water at three different temperatures: 4 °C (typical temperature of refrigeration), 22 °C (room temperature), or 92 °C (within the range of suggested temperature for hot brewed coffee^26^). The refrigerator and room brews were brewed for 0, 4, 8, 12, and 24 h and the hot brews were brewed for ‘0’, 4, 10, 30, and 60 min, each corresponding to time points denoted as T1, T2, T3, T4, and T5, respectively. Time points were chosen based on representative extraction stages of full immersion brews: T1 was the “rinsed” coffee, T2 and T3 were during and near finish of the rapid increase in *TDS*, and T4 and T5 were after the *TDS* of the brew had reached apparent equilibrium^19^.

All coffees were brewed using a full immersion brewing technique in Toddy Cupping Kits (Toddy LLC., Loveland, CO, USA) with Nestlé Pure Life Purified Water^27^ (Nestlé Waters North America Inc., Stamford, CT, USA) at a water-to-coffee brew ratio ${R}_{brew}=5$, which is a typical brew ratio the industry uses for cold brew concentrate^28^. For the refrigerator temperature brews, water was pre-refrigerated to 4 °C; for the room temperature brews, water temperature was set at 22 °C; for the hot brews, water was heated to 92 °C using a Bonavita 1.7-L Variable Temperature Electric Kettle (Bonavita World, Woodinville, WA, USA). All coffees were brewed using the following full immersion technique: First, 100 g of coffee grounds were measured and placed into the glass container, and 500 g of water at target temperatures (4, 22, or 92 °C) was poured into the beaker using a goose-neck kettle in circular pouring motion. The timing of the brew started upon completion of the water pouring (time = 0). Then, with the only exception of the “rinsed” coffee which was immediately filtered, the coffee brews were stirred with a stirring stick for 15 s to ensure all coffee grounds were fully wet. After that, the brews were covered with a lid, and brewed to the designated brew time. During brewing, the fridge brews were stored in a refrigerator at 4 °C, while the room and hot brews were placed on a wooden countertop at room temperature. When the brew reached the designated brew time, a filtration step was applied, in which the entire coffee brew was filtered through a filter paper to separate the liquid coffee brew from the coffee grounds. Two identical brews were carried out per brewing condition. The resulting coffee liquid brews from the identical brews were combined into a coffee concentrate, in which a 2 mL sample was drawn using a disposable pipet for *TDS* measurements. Then, the coffee concentrate was diluted with room temperature water by a factor of 2 by mass to obtain the diluted coffee beverage. A 100 mL sample was collected from the coffee beverage for physical/chemical measurements, and the rest of the diluted coffee beverage was stored at 4 °C overnight before sensory evaluation the next day. All coffee types were brewed in triplicate.

### Physical and chemical measurements

The total dissolved solids (*TDS*) were measured for both the coffee concentrate before dilution and the coffee beverage after dilution at room temperature using a VST LAB Coffee III digital refractometer (VST Inc, Boston, MA, USA). The refractometer was calibrated according to procedures outlined in a prior study^19^. The corresponding extraction yield, *E*, was calculated form the measured *TDS* and ${R}_{brew}$ using the following equation^19^:

1 $$E =\frac{TDS}{1-TDS}{R}_{brew}.$$

The pH and titratable acidity of the coffee beverage after dilution were also measured at room temperature using a Mettler Toledo S220 SevenCompact™ Benchtop pH/IS*E* Meter (Mettler Toledo, Greifensee, Switzerland). The pH measurements were taken while the sample was stirred using a magnetic stir bar. Then, while the coffee was stirred, the titratable acidity was measured by adding 0.1 M NaOH (Sigma Aldrich, St. Louis, MO, USA) dropwise from a burette, until the solution reached a pH of 8.20 ± 0.01. Titratable acidity is expressed in mL NaOH/50 mL coffee. The pH meter was calibrated using acidic (pH 4.00 ± 0.01, color coded red), neutral (pH 7.00 ± 0.01, color coded yellow), and basic (pH 10.00 ± 0.01, color coded blue) calibration standards (VWR chemicals, VWR international, Radnor, PA, USA) before every set of six measurements. Physical/chemical measurements were conducted in triplicate, consistent with the samples tasted in descriptive analysis.

### Descriptive analysis

The training and sensory evaluation protocols were adapted from a generic descriptive analysis in accordance with approved amended IRB protocol 1082569-2. The panel was composed of 14 panelists, including 10 women and 4 men. In the Fall of 2021, ballot development and training were conducted in a group setting over nine 1-h training sessions, where panelists tasted the coffees and engaged in free term generation, drawing inspiration from the Coffee Taster's Flavor Wheel^29^. Following the selection of 27 attributes, panelists engaged in additional discussions and training to come to consensus and familiarize themselves with reference standards for all attributes incorporated in the final ballot. During the last week of training, panelists received reference materials alongside coffee samples, allowing them to work directly with the references while tasting coffees. All the attributes on the ballot are listed along with reference standards in Supplementary Table S1. Before the actual evaluations, panelists conducted two mock sessions with a subset of the coffees to ensure alignment among panelists.

For the actual sensory evaluation, panelists participated in 15 tasting sessions, assessing 6 coffees per session. The evaluations were conducted in temperature-controlled, red-lit sensory booths to ensure panelists were isolated and not influenced by visual differences among the coffees. Samples were assigned a 3-digit random code and presented in a randomized Williams Latin Square block design to mitigate carryover effects. Coffees were presented to panelists one at a time, with a 2-min interval between samples. Data collection was conducted on RedJade (RedJade, Redwood City, CA, USA), where participants rated each attribute on an unstructured line scale ranging from 0 to 100, with anchor points at 10 and 90 labeled only with the words “Low” and “High”. Each coffee was evaluated in triplicate by the panelists on separate days. Coffee samples (50 mL) were served in white ceramic mugs. To maintain a consistent consumption temperature for all panelists, coffee samples were served cold around 6 °C and gradually warmed to around 12 °C within 3 min during the tasting. Unsalted saltine crackers, water, and an empty cup for expectoration were provided to all panelists.

### Data analyses

Descriptive analysis data were exported from the data collection software, and ratings on the unstructured line scales were converted to scores from 0 to 100 for each attribute. All statistical analyses were conducted using RStudio 2022.07.1 Build 554. To understand the sensory characteristics of the coffee samples, Multivariate Analysis of Variance (MANOVA) across all attributes, and Analysis of Variance (ANOVA) of individual attributes were used to determine which attributes were significantly different across the set of coffees. A three-way MANOVA (Product, Judge, Replication as factors) with two-way interactions using Type III sums of squares (SS) was used to analyze all the attributes at 5% significance level of alpha. The Wilk’s Lambda test was used in MANOVA. Following a significant MANOVA result, univariate three-way ANOVAs with two-way interactions on each attribute were conducted with Type III sums of squares at the same significant level. For any attribute, if the main product effect was significant, and a Product*Judge or Product*Replication effect was significant in the univariate ANOVA, a pseudo-mixed ANOVA using the mean square of the corresponding interaction effect as the error term was used to test the main product effect. Then, a five-way MANOVA (Roast, Origin, Temperature, Judge, Replication as factors) with up to three-way interactions using Type III sums of squares (SS) was used to analyze all the significant attributes at the same significance level. Attribute means determined to be significant for the experimental factors, including the ones from judge or replication effect by pseudo-mixed ANOVA, were then compared using Fisher’s Least Significant Difference (LSD) in the “agricolae” package.

Principal Component Analysis (PCA) based on the significant attribute means was used to visualize the sensory profiles of the 30 coffee samples. A correlation PCA was conducted using the “SensomineR” package, and a PCA biplot was created using the “factoextra” package. The first three dimensions were investigated and interpretations were focused on the first two dimensions. In addition, a correlation PCA based on the raw attribute ratings was conducted using Bootstrap techniques to display 95% confidence ellipses around products using the “SensoMineR” package. Pairwise comparisons of the products using Hotelling’s T2 tests were also generated by the same function.

Correlation analysis between the intensity of sensory attributes and physical/chemical measurements was conducted, and the corresponding correlation plot was generated using the “corrplot” package. Furthermore, Multiple Factor Analysis (MFA) was conducted based on means of significant attribute intensities using Time Point as groups to visualize the sensory profiles over time. The MFA compromise space was generated using the “FactoMineR” package, and the score plot and loading plot were generated using the “factoextra” package.

## Results

### Physical and chemical measures

**Figure 2:**
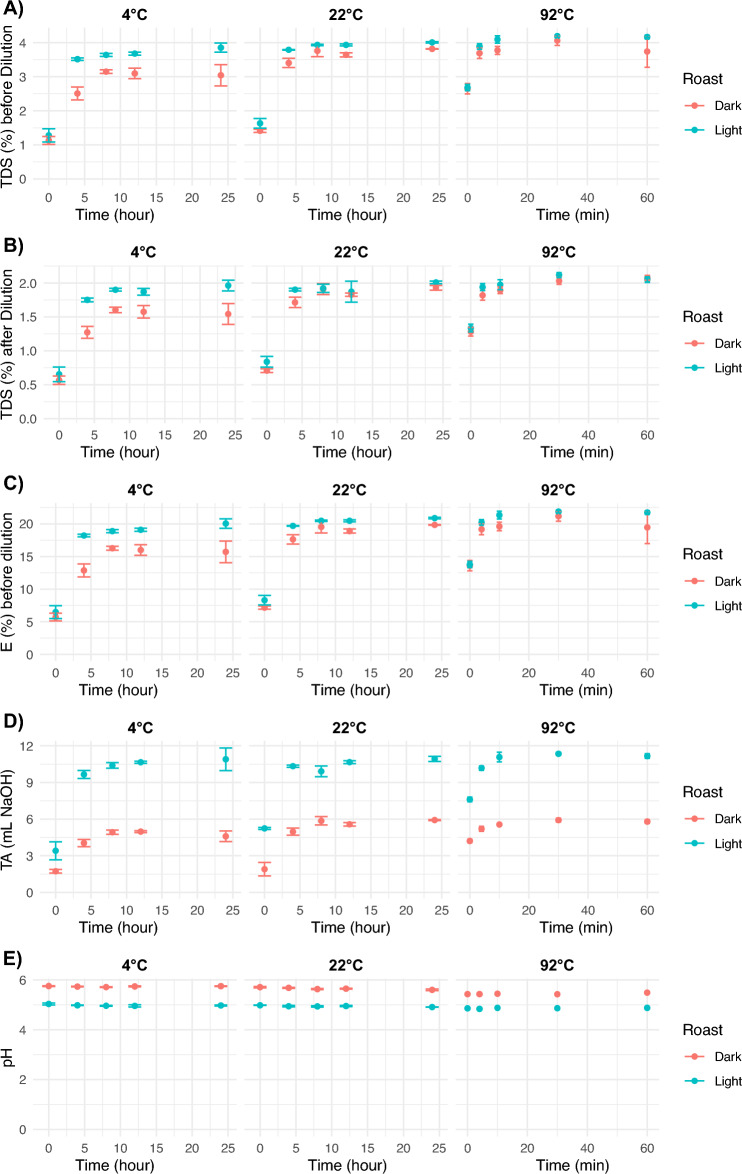
Scatter plot of the means and standard deviations of (**A**) TDS before dilution, (**B**) TDS after dilution, (**C**) Extraction before dilution, (**D**) Titratable Acidity, and (**E**) pH over time; roast levels are represented with different colors.

Overall, the *TDS* before and after dilution, extraction, and titratable acidity increased as brew time increased as shown in Fig. 2. All coffees were brewed to set times, which corresponded to target timepoints dependent on temperature. The 92 °C brews reached higher equilibrium *TDS* before dilution than the 22 °C and 4 °C brews, with averages of ($3.97\pm 0.63)\%$, ($3.93\pm 0.10)\%$, and ($3.52\pm 0.59)\%$ at the final timepoint, respectively. Consistent with previous studies^18^, roast level also affected the extraction dynamics, with the light roast being extracted faster and reaching higher *TDS* values than the dark roast at every time point, with this effect being the most pronounced for the 4 °C brews. Coffee brews were diluted by a factor of two for sensory evaluations and chemical measurements. Thus, the *TDS* after dilution and calculated extraction yield based on the concentrated *TDS* followed the same trends as the *TDS* before dilution.

The titratable acidity of the coffee samples varied significantly for roast, temperature, and brew time, while the pH only varied significantly with roast and temperature (cf. Fig. 2D,E). The light roast coffee had significantly higher titratable acidity and lower pH. Since titratable acidity is correlated with perceived sourness^7^, our results indicated that the light roast was more sour/acidic than the dark roast regardless of brewing temperature or brew time. The effect of temperature was smaller compared to that of roast; overall, the titratable acidity increased, and pH slightly decreased with brewing temperature. Furthermore, the titratable acidity of the coffee increased with brew time, but pH remained stable as brewing proceeded.

### Sensory measures

**Figure 3:**
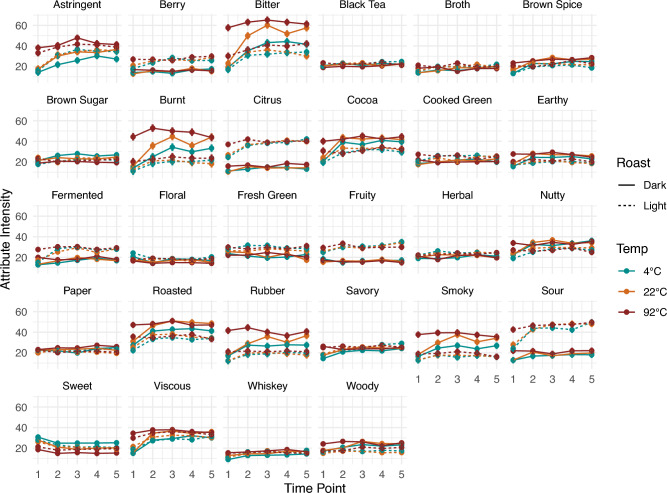
Line plots of mean attribute Intensities over time points of light and dark roasts. Time points 1, 2, 3, 4, 5 correspond to 0, 4, 8, 12, 24 h for 4 °C and 22 °C brews, and 0, 4, 10, 30, 60 min for 92 °C brews. Dark roast samples are represented with solid lines, and light roast samples are represented with dashed lines. Different brewing temperatures are represented with different colors, error bars represent the LSD values for each attribute from the 3-way ANOVA with interactions.

The three-way MANOVA with two-way interactions across all attributes showed that the main product (coffee samples) effect was significant. In addition, Judge, Rep, and two-way interactions also were significant (p-value < 0.05). Following the significant MANOVA, univariate three-way ANOVAs with two-way interactions for each attribute found that 25 out of the 28 attributes in the scorecard (all attributes except black tea, paper, and herbal) differed significantly among the 30 coffee samples (cf. Supplementary Table S2), indicating a wide range of sensory profiles within this product set. For comparison, the line plots of perceived attribute intensities of all attributes for both roast levels and brewed at three different temperatures over the different time points are shown in Fig. 3. Within this product set, some attributes' intensities varied largely with the experimental factors tested, such as, astringent, bitter, burnt, citrus, cocoa, roasted, rubber, smoky, and sour; while some attributes' intensities remained within a smaller range with respect to the change of experimental factors, such as broth, brown spice, brown sugar, cooked green, earthy, floral, savory, and whiskey.

Furthermore, the five-way MANOVA examining the effects of experimental factors showed that roast level was the largest driver of differences among the samples, followed by brewing temperature, then brew time, especially the longer brew times as *TDS* plateaued. Fourteen attributes were found to be significant for all three experimental factors—roast, temperature, and time (i.e., bitter, sour, cocoa, berry, citrus, rubber, nutty, roasted, fermented, burnt, smoky, earthy, and brown spices) (cf. Table 1). Additionally, fruity, cooked green, and fresh green were significantly different between roasts; sweet, savory, viscous, astringent, and whiskey were significantly different both among temperatures and across times; and brown sugar differed significantly among temperatures. Notably, many of the significant attributes by roast, temperature, or time showed significant two-way interaction(s) among the three experimental factors.

**Table 1:**
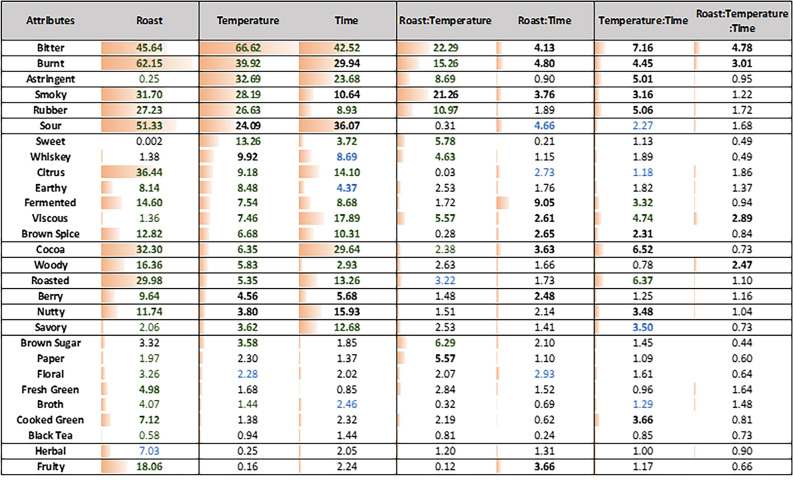
The F-values of the experimental factors (Roast, Temperature, Time) in the five-way ANOVAs of the sensory attributes as response variable. Established significance at 5% are indicated in bold; F-values calculated by pseudo-mixed ANOVA with Judge are in green, F-values calculated by pseudo-mixed ANOVA with Repetition are in blue.

### Relation between sensory and physical/chemical measures

**Figure 4:**
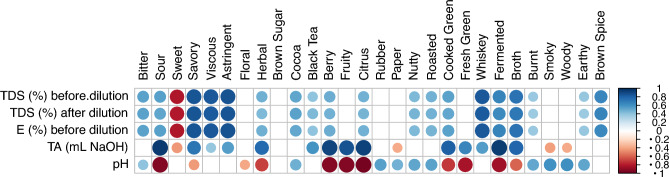
Correlations between physical/chemical measurements and attribute intensities. The absolute values of the correlation coefficients are reflected by the size of the circles. Significant positive correlations are indicated by blue circles, significant negative correlations are indicated by red circles, and the non-significant correlations are left blank.

Because the *TDS* and *E* are highly dependent on brew time, the relationship between the attribute intensities and *TDS* was investigated. Correlation analyses revealed that 19 attributes were positively correlated to *TDS* and E, while sweetness was the only attribute negatively correlated with *TDS* and E, and the remaining 8 attributes showed no statistically significant relationship with *TDS* or *E* (cf. Fig. 4). These findings suggest that most attribute intensities increased with *TDS* as brewing proceeded. Furthermore, correlation analyses between attribute intensities and chemical measurements were also conducted. Sour, berry, fruity, citrus, and fermented had the strongest positive correlations with titratable acidity, and negative correlation with pH, indicating those attributes were highly related to the acid content of the coffees.

### Effects of roast, temperature, and time

The 5-way ANOVA results show that the 17 attributes were significantly different by roast, as the largest driver of difference among the 30 coffee samples (Table 1). Roast level had the biggest effect on the following attributes in order of magnitude—burnt, sour, bitter, citrus, cocoa, smoky, roasted, rubber, and fruity, as indicated by decreasing F-values (Table 1). Overall, dark roast coffee brews had significantly higher bitter, burnt, smoky, roasted, rubber and cocoa intensities, while the light roast coffee brews were significantly more sour, citrusy, berry-like, and fruity (cf. Fig. 3), which is consistent with prior works^18,30^. Notably, fruity was only significant for roast, suggesting that the fruity flavor of the coffee was predominantly determined by roast level with light roast being significantly fruitier regardless of the brewing process.

**Figure 5:**
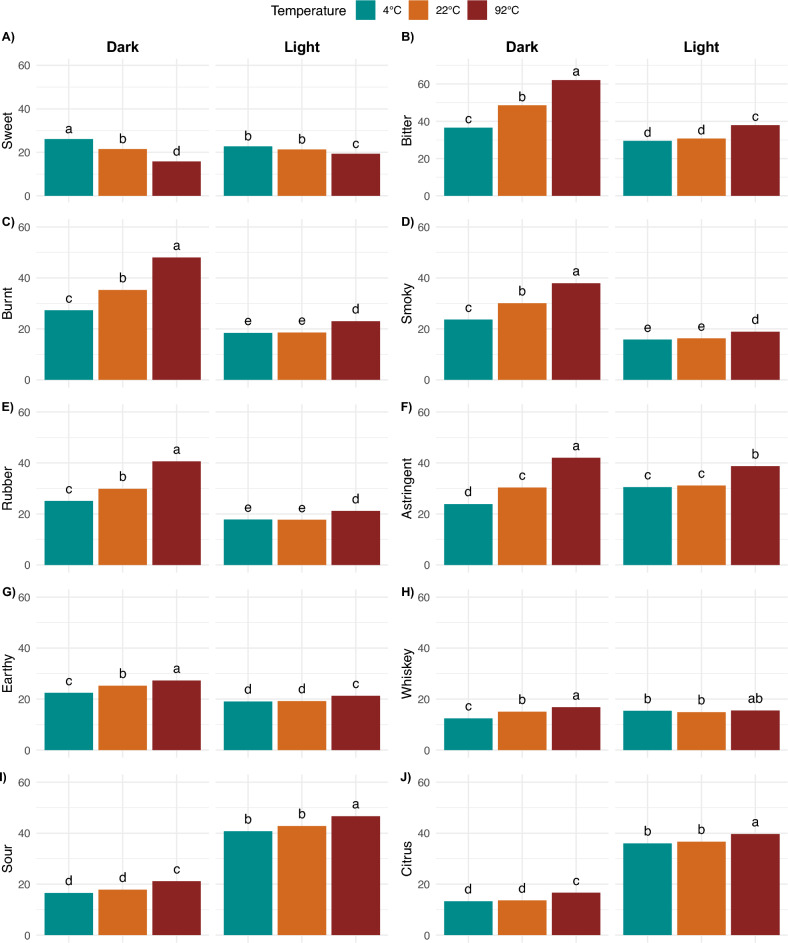
Sensory attributes with significant differences by temperature and roast level across all time points, with different letter codes indicating significant difference.

Temperature was the second largest driver of difference of the three experimental factors, with 20 attributes significantly different by temperature from the 5-way ANOVA. Similarly, bitter, burnt, smoky, rubber, sour; additionally, astringent, sweet, whiskey, citrus and earthy were also important attributes that varied by temperature (cf. Table 1). Sweetness significantly increased with decreasing temperature in dark roast, and the sweetness in the 92 °C light roast was significantly lower than in the 4 or 22 °C brews (cf. Fig. 5A). In contrast, sour and citrus were significantly higher in the 92 °C brews for both roast levels, due to the low perceived flavor intensities in the “rinsed” coffee at 22° and 4 °C (cf. Fig. 5I,J). Furthermore, many of the differences by temperature also depended on the roast. For dark roast, bitter, burnt, smoky, rubber, astringent, whiskey, and earthy notes significantly increased with brewing temperature from 4 °C, 22 °C, to 92 °C, while the changes with respect to temperature in light roast were not significant between the 4 °C and 22 °C brews (cf. Fig. 5B–H). These attribute intensities were significantly lower than 92 °C brews, except for whiskey, for the same reason that the “rinsed” coffee at 22 °C and 4 °C were lacking those flavors.

**Figure 6:**
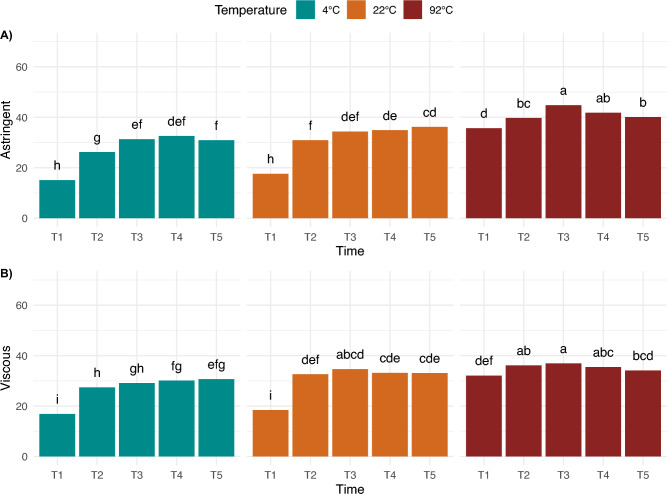
Sensory attributes with significant differences by temperature and time point across all roast levels, with different letter codes indicating significant difference.

**Figure 7:**
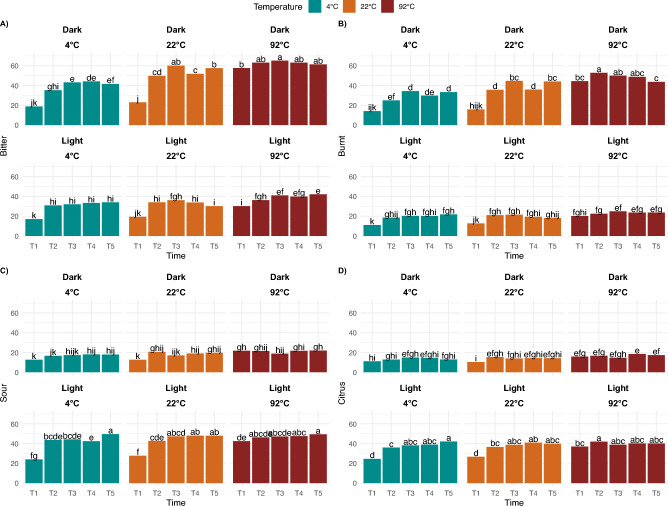
Sensory attributes with significant differences by roast level, temperature, and time point, with different letter codes indicating significant difference.

Surprisingly, the effect of time was the smallest within the experimental factors tested, and under our experimental brewing protocol, in which diluted coffee concentrates were evaluated. We therefore note that the effect of brew time could have been diminished compared to a typical coffee cupping or French-press method with a higher brew ratio. Within our experimental sample set then, Time Point 1 “rinsed” coffee was sweeter, while the other significant attributes had lower intensities. Interestingly, astringent and viscous, as mouthfeel and/or chemesthesis attributes, were only significant for temperature and time. Regardless of roast level, at 4 °C and 22 °C, both astringency and viscous mouthfeel generally increased with time (cf. Fig. 6). Despite the fact the “rinsed” coffee was significantly lower in intensity than all the other brews, the fridge and room brews for 8, 12, and 24 h were significantly more astringent than the 4-h brew, and the 12- and 24-h fridge brews were significantly more viscous than the 4-h brew. At 92 °C, the “rinsed” coffee was significantly lower in both viscous mouthfeel and astringency compared to the other timepoints. Furthermore, the effect of time was more pronounced at lower brewing temperature, but it also depended on roast level for specific attributes (cf. Fig. 7). For example, for dark roast, the changes in bitter and burnt attributes were significant over time in fridge and room brews, while the changes in hot brews remained relatively stable. Conversely, sour and citrus had more pronounced differences for light roast at lower temperature brews, such that the Light—4 °C—24 h brew had significantly higher sour and citrus notes compared to the Light—4 °C—4 h brew.

### Principal component analysis (PCA)

**Figure 8:**
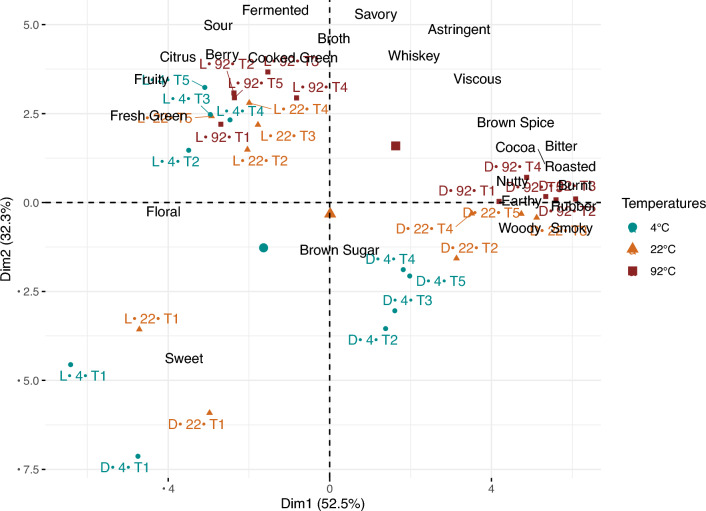
Principal component analysis biplot of coffee samples and significant sensory attributes from 3-way ANOVA. Different brewing temperatures are indicated by different colors. Nomenclature of the coffee samples are represented by: Roast (L: light roast/D: dark roast)—Temperature (4/22/92 °C)—Time Point (T1/T2/T3/T4/T5). Time points T1, T2, T3, T4, T5 corresponds to 0, 4, 8, 12, 24 h in 4 °C and 22 °C brews, and 0, 4, 10, 30, 60 min in 92 °C brews. See Supplementary Fig. 1 for important contributors to the first two PCs.

The correlation PCA biplot of the 25 significant attributes across 30 coffee samples is shown in Fig. 8. The first two dimensions explained 85% of the total variance, and the third dimension explained another 6%. These results accord with the 5-way MANOVA, as the coffee samples were separated the most by roast level. More specifically, attributes typically associated with dark roasts, for example, burnt, smoky, roasted, rubber, and bitter, contributed to the first dimension; and other attributes including fermented, savory, broth, sour, were important contributors to the second dimension of the PCA (cf. Supplementary Fig. S1). Additionally, the 0-min “rinsed” coffees at 4 °C and 22 °C were very different from all the other brews with much higher sweet and floral intensities and lack of other flavors.

The PCA further demonstrates that the effect of brewing temperature depended on roast level. For dark roast coffee brews, there was a separation by temperature, such that the 4 °C brews were higher in sweet, and floral intensities, 92 °C brews were more bitter, burnt, smoky, and astringent, and the 22 °C brews were somewhere in between. For light roast coffee, the separation of samples among the different temperatures was less clear on the PCA biplot. Similarly, the effect of brew time also depended on brewing temperature. In addition to the 0-min “rinsed” coffee at 4 °C and 22 °C being unique compared to all the other samples, clearer separations among coffees at lower brewing temperature were seen. More specifically, the dark roast coffees brewed at 4 °C exhibited some characteristics of shorter brews, and were sweeter and less bitter, roasted, or burnt. However, 92 °C coffee brews, at a given roast level, were very similar to each other.

Interestingly, for the light roast, the 92 °C “rinsed” coffee (L-92-T1) was very close to some of the 4 °C and 22 °C brews, such as samples L-4-T3 and L-4-T4, as shown in the PCA biplot. The p-values from multivariate pairwise comparisons between the coffee samples, except the “rinsed” coffee at 4 and 22 °C, are shown in Table 2. The Hotelling’s T2 test results revealed that, for light roast coffee, the sensory profiles of these long-time cold brews and short-time hot brews were not significantly different from each other, suggesting that the sensory profiles of these coffees were similar. However, this finding was roast dependent, and for dark roast, the short-time hot brews were significantly different from almost all of the long-time cold brews.

**Table 2:** p-values of the Hotelling’s T2 tests for pairwise comparison of sensory profiles between (A) light roast and (B) dark roast coffee samples. “t = 0” coffees brewed at 4 °C and 22 °C were excluded from the analysis. Significance at 5% is indicated in bold.

(A)	Light-4C-T2	Light-4C-T3	Light-4C-T4	Light-4C-T5	Light-22C-T2	Light-22C-T3	Light-22C-T4	Light-22C-T5	Light-92C-T1	Light-92C-T2	Light-92C-T3	Light-92C-T4	Light-92C-T5
Light-4C-T2	1.0E+00	3.4E−01	4.5E−01	**3.2E**−**03**	3.6E−01	**9.7E**−**03**	**4.7E**−**02**	5.0E−02	8.0E−01	**9.0E**−**04**	**1.5E**−**04**	**7.3E**−**05**	**7.0E**−**05**
Light-4C-T3	3.4E−01	1.0E+00	5.3E−01	8.8E−02	6.9E−01	1.7E−01	3.5E−01	4.8E−01	5.0E−01	**2.5E**−**02**	**2.2E**−**03**	**1.2E**−**03**	**1.2E**−**03**
Light-4C-T4	4.5E−01	5.3E−01	1.0E+00	**2.0E**−**03**	9.8E−01	5.0E−02	6.9E−02	**2.2E**−**02**	7.7E−01	**2.8E**−**03**	**3.1E**−**04**	**2.8E**−**04**	**5.0E**−**04**
Light-4C-T5	**3.2E**−**03**	8.8E−02	**2.0E**−**03**	1.0E+00	**2.9E**−**02**	3.4E−01	7.6E−01	4.6E−01	**9.1E**−**04**	2.1E−01	**1.3E**−**02**	**1.3E**−**03**	**2.6E**−**03**
Light-22C-T2	3.6E−01	6.9E−01	9.8E−01	**2.9E**−**02**	1.0E+00	1.2E−01	1.6E−01	1.5E−01	6.8E−01	**1.7E**−**02**	**1.6E**−**03**	**1.3E**−**03**	**1.5E**−**03**
Light-22C-T3	**9.7E**−**03**	1.7E−01	5.0E−02	3.4E−01	1.2E−01	1.0E+00	7.7E−01	4.3E−01	**1.2E**−**02**	6.6E−01	6.2E−02	**3.1E**−**02**	**4.8E**−**02**
Light-22C-T4	**4.7E**−**02**	3.5E−01	6.9E−02	7.6E−01	1.6E−01	7.7E−01	1.0E+00	7.3E−01	5.7E−02	6.1E−01	5.0E−02	**1.1E**−**02**	**2.6E**−**02**
Light-22C-T5	5.0E−02	4.8E−01	**2.2E**−**02**	4.6E−01	1.5E−01	4.3E−01	7.3E−01	1.0E+00	**2.6E**−**02**	1.8E−01	1.4E−02	**5.4E**−**03**	**8.8E**−**03**
Light-92C-T1	8.0E−01	5.0E−01	7.7E−01	**9.1E**−**04**	6.8E−01	**1.2E**−**02**	5.7E−02	**2.6E**−**02**	1.0E+00	4.7E−04	7.8E−05	**4.3E**−**05**	**6.8E**−**05**
Light-92C-T2	**9.0E**−**04**	**2.5E**−**02**	**2.8E**−**03**	2.1E−01	**1.7E**−**02**	6.6E−01	6.1E−01	1.8E−01	**4.7E**−**04**	1.0E+00	1.4E−01	**4.3E**−**02**	1.0E−01
Light-92C-T3	**1.5E**−**04**	**2.2E**−**03**	**3.1E**−**04**	**1.3E**−**02**	**1.6E**−**03**	6.2E−02	5.0E−02	**1.4E**−**02**	**7.8E**−**05**	1.4E−01	1.0E+00	7.4E−01	9.3E−01
Light-92C-T4	**7.3E**−**05**	**1.2E**−**03**	**2.8E**−**04**	**1.3E**−**03**	**1.3E**−**03**	**3.1E**−**02**	**1.1E**−**02**	**5.4E**−**03**	**4.3E**−**05**	4.3E−02	7.4E−01	1.0E+00	6.1E−01
Light-92C-T5	**7.0E**−**05**	**1.2E**−**03**	**5.0E**−**04**	**2.6E**−**03**	**1.5E**−**03**	**4.8E**−**02**	**2.6E**−**02**	**8.8E**−**03**	**6.8E**−**05**	1.0E−01	9.3E−01	6.1E−01	1.0E+00

(B)	Dark-4C-T2	Dark-4C-T3	Dark-4C-T4	Dark-4C-T5	Dark-22C-T2	Dark-22C-T3	Dark-22C-T4	Dark-22C-T5	Dark-92C-T1	Dark-92C-T2	Dark-92C-T3	Dark-92C-T4	Dark-92C-T5
Dark-4C-T2	1.0E+00	1.2E−01	1.2E−01	2.9E−01	**7.1E**−**03**	**2.2E**−**04**	**5.7E**−**04**	**1.2E**−**04**	**1.6E**−**05**	**4.5E**−**07**	**1.3E**−**06**	**4.7E**−**07**	**4.7E**−**05**
Dark-4C-T3	1.2E−01	1.0E+00	7.0E−01	6.8E−01	**3.1E**−**03**	**3.1E**−**03**	**6.4E**−**03**	**1.0E**−**03**	**1.9E**−**05**	**2.9E**−**07**	**3.9E**−**06**	**2.2E**−**07**	**2.4E**−**05**
Dark-4C-T4	1.2E−01	7.0E−01	1.0E+00	7.7E−01	**3.4E**−**02**	**2.1E**−**03**	**4.7E**−**03**	**5.6E**−**04**	**5.0E**−**05**	**5.8E**−**07**	**1.7E**−**06**	**8.3E**−**07**	**1.9E**−**04**
Dark-4C-T5	2.9E−01	6.8E−01	7.7E−01	1.0E+00	1.1E−01	**1.3E**−**03**	**4.1E**−**03**	**7.6E**−**04**	**5.3E**−**04**	**9.9E**−**06**	**4.1E**−**06**	**1.9E**−**05**	**1.1E**−**03**
Dark-22C-T2	**7.1E**−**03**	**3.1E**−**03**	**3.4E**−**02**	1.1E−01	1.0E+00	**2.4E**−**03**	**1.9E**−**02**	**4.8E**−**03**	**1.0E**−**02**	**7.6E**−**05**	**2.1E**−**05**	**1.8E**−**04**	**1.7E**−**02**
Dark-22C-T3	**2.2E**−**04**	**3.1E**−**03**	**2.1E**−**03**	**1.3E**−**03**	**2.4E**−**03**	1.0E+00	1.8E−01	4.1E−01	**4.5E**−**02**	**2.0E**−**03**	**1.9E**−**02**	**3.4E**−**04**	**5.7E**−**03**
Dark-22C-T4	**5.7E**−**04**	**6.4E**−**03**	**4.7E**−**03**	**4.1E**−**03**	**1.9E**−**02**	1.8E−01	1.0E+00	4.9E−01	**3.6E**−**02**	**5.0E**−**04**	**2.0E**−**03**	**3.7E**−**04**	**1.9E**−**02**
Dark-22C-T5	**1.2E**−**04**	**1.0E**−**03**	**5.6E**−**04**	**7.6E**−**04**	**4.8E**−**03**	4.1E−01	4.9E−01	1.0E+00	1.7E−01	**5.3E**−**03**	**2.6E**−**02**	**2.0E**−**03**	**4.7E**−**02**
Dark-92C-T1	**1.6E**−**05**	**1.9E**−**05**	**5.0E**−**05**	**5.3E**−**04**	**1.0E**−**02**	**4.5E**−**02**	**3.6E**−**02**	1.7E−01	1.0E+00	2.7E−01	3.7E−01	1.3E−01	6.7E−01
Dark-92C-T2	**4.5E**−**07**	**2.9E**−**07**	**5.8E**−**07**	**9.9E**−**06**	**7.6E**−**05**	**2.0E**−**03**	**5.0E**−**04**	**5.3E**−**03**	2.7E−01	1.0E+00	5.3E−01	2.8E−01	7.3E−01
Dark-92C-T3	**1.3E**−**06**	**3.9E**−**06**	**1.7E**−**06**	**4.1E**−**06**	**2.1E**−**05**	**1.9E**−**02**	**2.0E**−**03**	**2.6E**−**02**	3.7E−01	5.3E−01	1.0E+00	**2.6E**−**02**	2.5E−01
Dark-92C-T4	**4.7E**−**07**	**2.2E**−**07**	**8.3E**−**07**	**1.9E**−**05**	**1.8E**−**04**	**3.4E**−**04**	**3.7E**−**04**	**2.0E**−**03**	1.3E−01	2.8E−01	**2.6E**−**02**	1.0E+00	5.7E−01
Dark-92C-T5	**4.7E**−**05**	**2.4E**−**05**	**1.9E**−**04**	**1.1E**−**03**	**1.7E**−**02**	**5.7E**−**03**	**1.9E**−**02**	**4.7E**−**02**	6.7E−01	7.3E−01	2.5E−01	5.7E−01	1.0E+00

### Multiple factor analysis (MFA)

**Figure 9:**
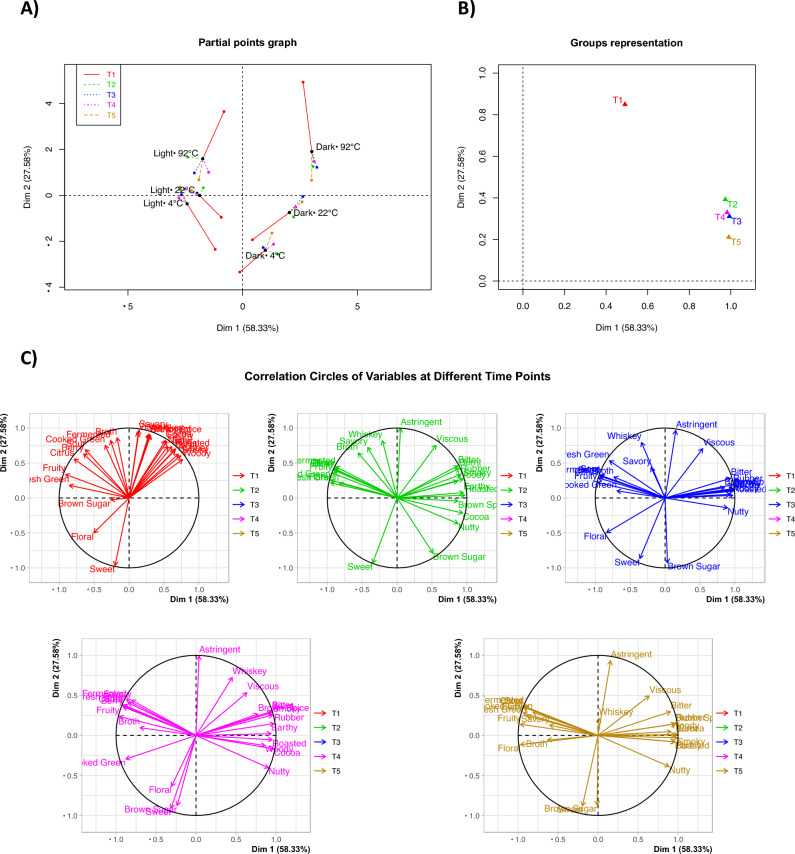
(**A**) Individual factor map with partial axes correlation plot, (**B**) group representation, and (**C**) correlation circles by group from MFA using time points (T1, T2, T3, T4, T5) as groups.

The MFA using time points as a reflection of extraction stages as groups is shown in Fig. 9. The first two dimensions accounted for 85.9% of the total variance, and the third dimension explained another 5.7%. Again, samples were separated primarily by roast level, then by brewing temperature, with the light roasts on the left and dark roast on the right side of the first dimension (cf. Fig. 9A). The partial axes plot also revealed that the differences by temperature also depended on the roast level. More specifically, light roast coffees brewed at 4 °C and 22 °C were closer to each other compared to the 4 °C and 22 °C brews from the dark roast, indicating that room and fridge brews using light roast coffee were more similar to each other compared to those brewed using the dark roast (cf. Fig. 9A). The MFA further demonstrates that light roast coffee samples across brew temperature and brew time were more similar to each other compared to the dark roast coffee samples, since they are positioned closer to each other with shorter partial axes. Thus, it is consistent with the multivariate pairwise comparison results that the sensory profiles of short hot brews and long cold brews were more similar in light roast coffee. Furthermore, the coffee samples from Time Point 1 was again the most different from the other time points, while the coffees brewed after the “rinsed” stage were much closer to each other (cf. Fig. 9B). Moreover, the correlation circles of variables at different time points also showed that the sensory profiles of this product set did not change very much after Time Point 1 (cf. Fig. 9C), indicating that longer brew times had a much smaller effect on the sensory profiles of full immersion brewed coffee compared to roast level and brewing temperature.

## Discussion

### The effect of brew time

Our analyses consistently found that brew time was the least substantial driver of sensory differences among the full immersion brewed coffee samples compared to roast level and temperature. Coffees from Time Point 1 were clearly different from the rest of the coffees regardless of roast or brewing temperature; yet, few statistically significant changes were observed in the sensory profiles of coffees brewed beyond Time Points 3 as *TDS* plateaued. Another reason why brew time had such a small effect possibly is that time points corresponding to each extraction stage as a factor were used instead of the actual continuous brew time as a parameter. While the variance within treatment is larger due to the natural difference in *TDS* within the same Time Point at different brewing temperatures (cf. Supplementary Fig. S2), it is harder to find differences comparing to the variance between treatments. Thus, there is more control over roast level and brewing temperature, but comparatively less control over the final *TDS* corresponding to different brew times at different brewing temperatures due to the natural difference in extraction dynamics of full immersion brews^16,19^.

Similarly, a previous fractionation study of the flavor profiles of drip brew coffee over time had also found strong correlations between attribute intensities and *TDS*, which was naturally imposed by brew time^6^. In the present study, we found that bitterness, sourness and astringency were positively correlated with *TDS*, whereas sweetness was negatively correlated to *TDS*. It is important to note that the coffee samples evaluated in full immersion brews were at their accumulative *TDS* from $t=0$ brewed up to a certain time point; in contrast, coffee samples evaluated in the drip brew study were at their instantaneous *TDS*, which corresponds to a fraction of a “whole brew”, in which each fraction was collected at 30 s increments of the brewing period. This suggests that *TDS*, rather than the actual brew time, acts as the factor driving flavor in both drip brew and full immersion brews. Interestingly, sweet and floral attributes were perceived with higher intensity towards the end of the drip brew, while sweetness was perceived as more intense at the beginning of full immersion brews, in both cases due to the lower *TDS* levels. Here, the perception of sweetness probably is more related to the impression of sweetness and the absence or low level of other flavors such as bitterness and acidity rather than actual perceived sweetness^4–6^. Additionally, the lack of a significant correlation between floral and *TDS* in this study might be due to the specific beans used, as floral aroma was not a significant attribute within the coffee sample set we tested, and coffee origin was found to be the second most influential factor in the variations among full immersion brewed coffees^4^. This observation suggests that the floral characteristics of the Central American blend used in this study might not have been pronounced, and other coffees may be impacted differently by brew time.

### Short-time hot chilled brews vs. long-time cold brews

An interesting finding is that the sensory profiles of certain short-time hot brews, especially the 92 °C “rinsed” coffee, were similar to those of long-time cold brews at 4 °C and 22 °C for the light roast coffee we used, indicating an overlap in sensory perception between short-time hot brew and long-time cold brew for the full immersion brewing technique. Yet, the extent of this overlap remains unclear and appears to be dependent on the roast level of the coffee, as this overlap was much more obvious in the particular light roast coffee we used in this study. To further validate this finding, discrimination testing will be needed to compare coffee samples brewed from long-time-cold-temperature and short-time-hot-temperature from a wider range of pre-brewing conditions including origins, processing methods, storage conditions, and roast levels.

Furthermore, other coffee brewing and coffee serving parameters also contribute to this overlap. In our experimental approach, we brewed the coffee concentrate and then diluted it by a factor of 2 the day prior to sensory evaluation, then the coffee beverages were chilled in the refrigerator before serving. Noted that this approach resulted in a lower brew ratio than a standard coffee cupping preparation protocol or a typical French press brewing method; in this manner we attempted to minimize the effect of varied serving temperature on sensory perceptions, by serving all coffee samples at a controlled, cold temperature. As a consequence of this approach, however, another potential confounding factor is that the overnight storage time might affect the chemical composition and sensory profile of the coffee, if it is stored at refrigerated temperature^31^. More detailed chemical analyses would be necessary to test this hypothesis.

### Practical implications

The overarching finding from our sensory analysis is that roast was the main driver of the overall flavor profile of full immersion brewed coffee, while temperature and time provided tools to optimize attribute intensities for the flavor profile of the roasted coffee beans we used. By examining the dynamic sensory profile of cold brews under different brewing parameters over time, our results not only strengthened the scientific understandings of existing common cold brew industry practices, but also provided insights for improving brew control and efficiency. For instance, fruity flavor, an important driver of consumer liking^1,2,4,5^, was only significant by roast level, suggesting that darker roasts may hinder the expression of fruitiness regardless of brewing temperature or brew time. Previous sensory analysis of full immersion coffee that examined the flavor profile of full immersion brews at fixed *TDS* found that the difference within the coffee sample was driven by roast level, origin, and then temperature^4^. Thus, when aiming to achieve specific sensory properties of coffee using full immersion brewing, it is crucial for practitioners to prioritize designing the roast of the coffee beans, followed by the temperature, and lastly, the longer brew times, especially after Time Point 3 as the *TDS* approaches equilibrium. When brewing light roast coffee, it is worth considering the possibility of achieving similar sensory profiles of long-time cold brews using short-time chilled hot brews through a controlled preparation method. Although similar flavor profiles can be obtained in a shorter time, the energy required for brewing water and chilling the coffee should be considered. From efficiency improvement aspects, a noteworthy implication is the potential for shorter brew times in crafting cold brew than conventionally assumed. The current general practice of cold brew falls between 12 to 24 h^12,28,32,33^. We observed very few statistically significant changes in the sensory profiles after Time Point 3 (8 h at 4 °C and 22 °C), suggesting that the industry can increase efficiency in full immersion styled cold brew coffee by reducing brew time without changing any other brewing conditions.

## Conclusions

Our study demonstrated the significant impact of roast level, temperature, and brew time on the sensory quality of full immersion brews. A wide range of flavor profiles were achieved from a single source green coffee blend through combination of these factors, with roast level as the biggest driver of flavor, followed by temperature, then the longer brew time points as *TDS* plateaued. Furthermore, perceived sweetness was negatively correlated with *TDS*, while 19 other attributes had a significant positive correlation with *TDS*. Interestingly, certain short-time hot brews, especially the 92 °C “rinsed” coffee, had similar sensory profiles to those of long-time cold brews at 4 °C and 22 °C for the light roast, although this similarity may depend on roast level and characteristics of the coffee beans used. These findings provide valuable understandings of the effects of coffee preparation factors on extraction and sensory profiles of full immersion coffee over time, and bring new practical insights into the control of sensory quality of full immersion brews for the coffee industry.

### Supplementary Information

Supplementary Information.

## Data Availability

All data generated and analyzed in this study are available at Dryad Digital Repository, 10.5061/dryad.v15dv423h.
