# Supplementary material for: Sensory analysis of the flavor profile of full immersion hot, room temperature, and cold brewed coffee over time
Source: Sci Rep. 2024 Aug 20;14:19298. doi: 10.1038/s41598-024-69867-6 (PMC11335879; doi:10.1038/s41598-024-69867-6)
Supplement: Supplementary file 1 — Supplementary Information. [file 41598_2024_69867_MOESM1_ESM.docx]

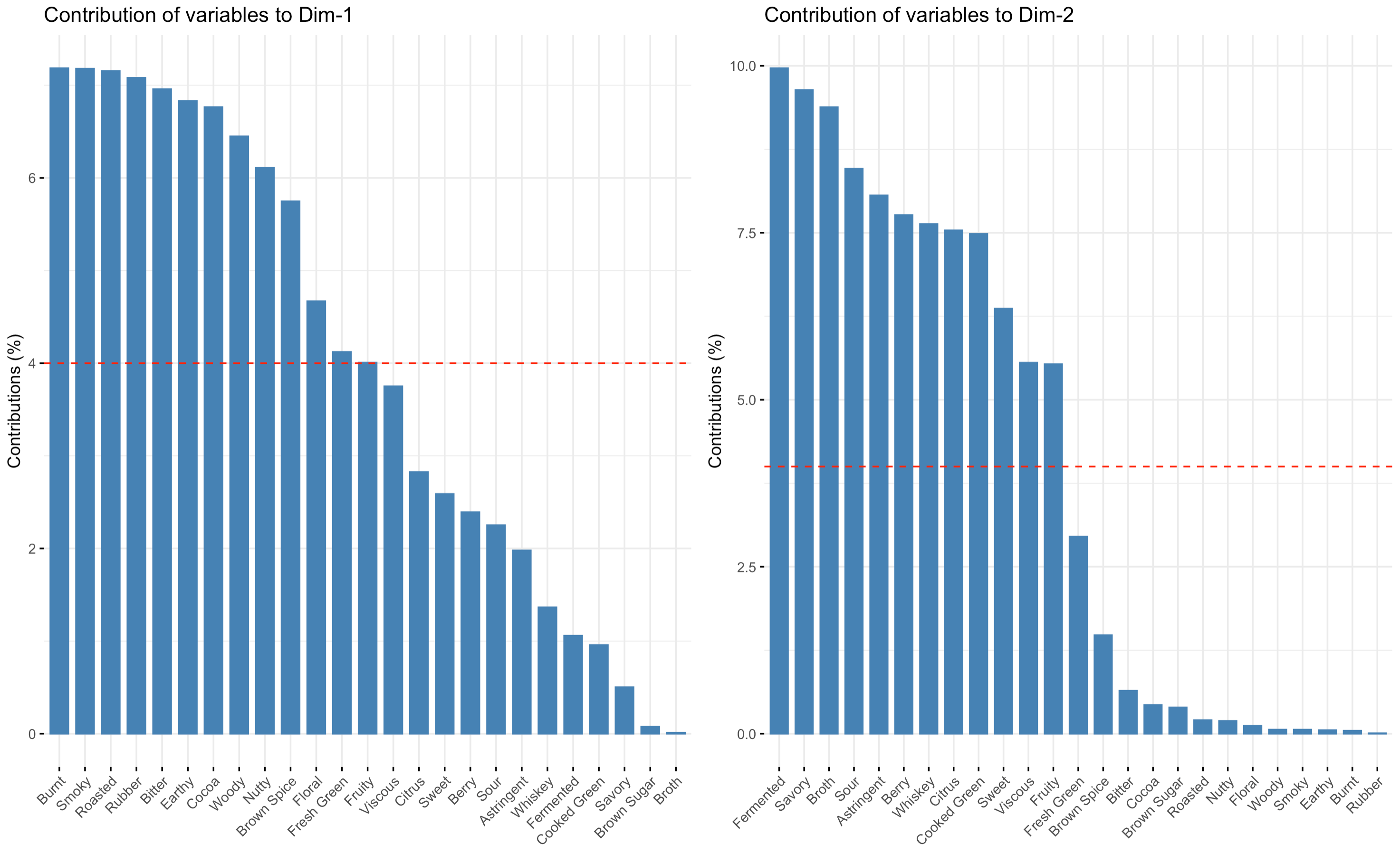


**Supplementary Figure S1.** Bar plots of the important contributors to PC 1 and PC 2 in the PCA. This dashed line corresponds to the expected value if the contributions were uniform.

**Supplementary Figure S2.** TDS after dilution by temperature and time point across roast levels, with different letter codes indicating significant difference.

| **Attributes** | **Reference Standards** |
| --- | --- |
| Bitter | Basic taste of bitter (0.1% caffeine solution in water) |
| Sour | Basic taste of sour (1.25% citric acid solution in water) |
| Sweet | Basic taste of sweet (2% sugar solution in water) |
| Savory | Basic taste of umami (1% MSG solution in water) |
| Viscous | Thick-It Clear Advantage Thickened Water |
| Astringent | Mouth-drying, puckering feeling (2% alum solution in water) |
| Floral | Dry Celestial Seasonings chamomile lavender tea |
| Herbal | Dried parsley |
| Brown Sugar | C&H Pure Cane Sugar, Golden Brown |
| Cocoa | Hershey's Unsweetened Cocoa Powder |
| Black Tea | Brewed Lipton black tea |
| Berry | mixed berries (blackberry, strawberry, raspberry, blueberry) |
| Fruity | Signature Select mixed fruit (White Grape Juice from Concentrate, Pineapple, Peaches, Pears, Cherry) |
| Citrus | Fresh sliced lemon and orange |
| Rubber | Cut pieces of bicycle inner tube |
| Paper | damp cardboard |
| Nutty | Justin’s almond butter |
| Roasted | Roasted barley |
| Cooked Green | Green Giant cut green beans |
| Fresh Green | Sliced green bell pepper and cucumber |
| Whiskey | Jack Daniels Tennessee Whiskey |
| Fermented | Guinness dark beer |
| Broth | Better than Bouillon roasted vegetable concentrate |
| Burnt | French Roast coffee |
| Smoky | Wright's Liquid Smoke |
| Woody | Twigs and Cedar balls |
| Earthy | Damp Miracle-Gro Potting Soil |
| Brown Spice | Equal parts cinnamon, nutmeg, clove, and allspice |

**Supplementary Table S1.** Reference standards used for the sensory attributes chosen by the panelists.

| Attribute | ANOVA | | Pseudo-mixed ANOVA using Product*Judge | | Pseudo-mixed ANOVA using Product*Rep | |
| --- | --- | --- | --- | --- | --- | --- |
|  | F value | p-value | F value | p-value | F value | p-value |
| **Bitter** | 48.1477 | < 2.2e-16 *** | **28.89** | **<2e-16 ***** | 34.82 | <2e-16 *** |
| **Sour** | 60.9065 | < 2.2e-16 *** | **32.97** | **<2e-16 ***** | 28.79 | <2e-16 *** |
| **Sweet** | 7.3594 | < 2.2e-16 *** | **3.267** | **8.92e-08 ***** | N/A | N/A |
| **Savory** | 5.2356 | < 2.2e-16 *** | **3.816** | **8.44e-10 ***** | N/A | N/A |
| **Viscous** | 16.1833 | < 2.2e-16 *** | **7.246** | **<2e-16 ***** | N/A | N/A |
| **Astringent** | 19.7316 | < 2.2e-16 *** | **10.19** | **<2e-16 ***** | N/A | N/A |
| **Floral** | 2.5905 | 1.211e-05 *** | **2.075** | **0.00115 **** | N/A | N/A |
| Herbal | 2.1729 | 0.0003898 *** | N/A | N/A | 1.486 | 0.0995 |
| **Brown Sugar** | 2.4186 | 5.234e-05 *** | **2.093** | **0.00101 **** | N/A | N/A |
| **Cocoa** | 14.0112 | < 2.2e-16 *** | **10.97** | **<2e-16 ***** | N/A | N/A |
| Black Tea | 0.9153 | 0.5958639 | - | - | - | - |
| **Berry** | 11.6807 | < 2.2e-16 *** | **6.248** | **<2e-16 ***** | N/A | N/A |
| **Fruity** | 18.5152 | <2e-16 *** | **8.967** | **<2e-16 ***** | N/A | N/A |
| **Citrus** | 59.4370 | < 2.2e-16 *** | **24.14** | **<2e-16 ***** |  |  |
| **Rubber** | 21.5944 | < 2.2e-16 *** | **13.69** | **<2e-16 ***** | N/A | N/A |
| Paper | 1.9136 | 0.002835 ** | 1.123 | 0.305 | N/A | N/A |
| **Nutty** | 7.2648 | < 2.2e-16 *** | **5.203** | **5.71e-15 ***** | N/A | N/A |
| **Roasted** | 20.4556 | < 2.2e-16 *** | **9.891** | **<2e-16 ***** | N/A | N/A |
| **Cooked Green** | 3.1679 | 6.818e-08 *** | **2.606** | **2.02e-05 ***** | N/A | N/A |
| **Fresh Green** | 5.4657 | < 2.2e-16 *** | **2.494** | **4.87e-05 ***** | N/A | N/A |
| **Whiskey** | 3.1173 | 1.088e-07 *** | **2.12** | **0.000833 ***** | N/A | N/A |
| **Fermented** | 15.4128 | < 2.2e-16 *** | **8.249** | **<2e-16 ***** | N/A | N/A |
| **Broth** | **2.5555** | **1.637e-05 ***** | N/A | N/A | N/A | N/A |
| **Burnt** | 31.0645 | < 2.2e-16 *** | **24.47** | **<2e-16 ***** | N/A | N/A |
| **Smoky** | 22.8506 | < 2.2e-16 *** | **14.47** | **<2e-16 ***** | N/A | N/A |
| **Woody** | 7.2134 | < 2.2e-16 *** | **4.522** | **1.96e-12 ***** | N/A | N/A |
| **Earthy** | 6.4763 | < 2.2e-16 *** | **4.486** | **2.67e-12 ***** | N/A | N/A |
| **Brown Spice** | 7.6666 | < 2.2e-16 *** | **5.168** | **7.69e-15 ***** | N/A | N/A |

**Supplementary Table S2.** F value and p-value of main product effect (Coffee) in three different ANOVAs for each attribute. Statistical significance at (p<0.05) is denoted by *. Attributes with significant main product effect are indicated in bold.
